# Cadmium-Induced Toxicity as a Pathophysiological Mechanism for Parkinson’s Disease Onset in Individuals with Iron and Zinc Deficiencies and Chronic Obstructive Pulmonary Disease

**DOI:** 10.3390/neurolint18060111

**Published:** 2026-06-04

**Authors:** Milan Aksic, Ana Cirovic, Orish Ebere Orisakwe, Vuk Djulejic, Bruna Puty, Rafael Rodrigues Lima, Aleksandar Cirovic

**Affiliations:** 1 Institute of Anatomy, Faculty of Medicine, University of Belgrade, Dr Subotica 4/2, 11000 Belgrade, Serbia; milan.aksic@med.bg.ac.rs (M.A.); ana.zekavica@med.bg.ac.rs (A.C.); vuk.djulejic@med.bg.ac.rs (V.D.); 2 Advanced Research Centre, European University of Lefke, TR-10, Mersin 99010, Turkey; 3 Laboratory of Functional and Structural Biology, Institute of Biological Sciences, Federal University of Pará, Augusto Corrêa Street, n. 01, Belém 66075-110, PA, Brazil; prof.bruna@finama.edu.br (B.P.); rafalima@ufpa.br (R.R.L.)

**Keywords:** Parkinson disease, cadmium, COPD, iron deficiency anemia, zinc deficiency

## Abstract

The pathophysiological basis of Parkinson’s disease (PD) remains incompletely understood. However, the influence of environmental factors, such as continuous cadmium exposure, requires further investigation. Notably, common comorbidities such as iron deficiency anemia (IDA), chronic obstructive pulmonary disease (COPD), and zinc deficiency are linked with increased cadmium bioavailability, and elevated blood cadmium levels have been reported in individuals with PD. Cd (II) deposits in the midbrain, causing the accumulation of inflammatory lipids, which promote neuronal destruction. Cd-treated animals develop Parkinson-like syndromes, and cadmium exposure is associated with neuronal loss and disruption of dopaminergic receptor expression. Neurofilament light chain (NfL), a biomarker of neurodegeneration, has been found to be elevated in patients with Parkinson’s disease and correlates with Cd blood concentrations. Iron deficiency promotes the secretion of FGF-23, which depletes vitamin D levels, further increasing the risk of PD. Moreover, COPD and IDA are two well-known examples of systemic hypoxia, which attracts metals bound to transferrin, such as cadmium and iron, leading to increased metal accumulation in various tissues, including the brain. Lead levels are also elevated in individuals with IDA, contributing to the risk of PD. Additionally, Cd exposure is associated with a reduced abundance of Lachnospiraceae in stool and decreased levels of butyrate, both of which are characteristic features of patients with Parkinson’s disease. Therefore, this review aims to explore how COPD, IDA, and zinc deficiency—known risk factors for Parkinson’s disease—lead to an increased cadmium burden and contribute to the onset and progression of the disease.

## 1. Introduction

Prevalence of Parkinson’s disease (PD) is estimated to be slightly less than 200 new cases per 100,000 individuals, making it one of the most common neurodegenerative disorders [1]. The etiology of PD is not fully understood, although some risk factors are identified. These include advanced age, male sex, brain trauma and exposure to environmental toxins such as some metals and pesticides, all of which are known to increase the risk of PD onset [2]. Therefore, PD is considered a multifactorial disease influenced by environmental factors in individuals with a suitable genetic background [3]. While some risk factors, such as age and male sex, are unchangeable, it is possible to reduce exposure to environmental factors like heavy metals, thereby decreasing the risk of PD.

Cadmium (Cd) is a heavy metal known for its neurotoxicity [4,5,6] and it has been associated with PD [7]. Exposure to Cd(II) leads to various neurological symptoms, including vertigo, headaches, and Parkinson-like symptoms [8,9]. Cd(II) is an environmental threat, since all types of food are contaminated with varying levels of this metal [10,11]. For example, rice, which is considered a major dietary source of Cd in many Far Eastern countries, readily binds cadmium due to thermodynamically favorable interactions between Cd and rice proteins [12]. It is absorbed in its divalent form. Several coordination sites are present on the surface of rice proteins, allowing Cd to bind primarily to cysteine, glutamic acid, aspartic acid, and histidine residues through interactions with carboxyl, hydroxyl, mercapto, and imidazole functional groups [13]. It is interesting that cooking and washing are ineffective in removing Cd from polluted food [14]. An average person consumes a few dozen micrograms of cadmium daily through diet alone [15,16]. Airborne cadmium levels are substantially higher in urban and industrialized areas when compared to rural zones [17], which means that cadmium exposure comes not only from contaminated food but also from polluted air. The World Health Organization has established 25 µg/kg as a tolerable monthly Cd intake limit in an adult individual [18]. However, individuals living in urban areas show blood Cd levels ranging from 0.22 to 0.27 µg/dL [19], indicating that individuals with Cd levels at the upper limit may reflect exposure above the safety threshold that could be associated with an increased health risk, such as PD. While this increased incidence cannot be entirely attributed to higher cadmium concentrations, cadmium exposure certainly plays a contributory role.

Desirable vitamin D levels are necessary not only for maintaining calcium homeostasis, but also for achieving the non-calciotropic effects of vitamin D. For example, inadequate vitamin D supplementation has been associated with poorer survival rates among individuals with malignancies [20,21] and with the onset of autoimmune diseases [22]. Moreover, reduced blood concentrations of vitamin D have been linked to multiple neurological disorders [23]. However, the potential effects of cadmium on the interaction between vitamin D levels and PD have not been discussed in detail.

Intestinal bacteria and the balanced relationship among microbial species are essential for the synthesis of numerous molecules important for human health, including short-chain fatty acids such as acetate and propionate, as well as certain vitamins, including vitamin K. Gut microbiota also play a crucial role in immune system function, while intestinal dysbiosis has been associated with various adverse health outcomes, including cancer [24], cardiovascular disease [25] and neurological disorders, such as Alzheimer’s [26,27], multiple sclerosis [28], and others. As humans are primarily exposed to Cd through the oral route, particularly non-smokers, gut bacteria inevitably interact with Cd. Consequently, Cd exposure may alter gut microbiota composition and balance. However, whether Cd induces changes in gut microbiota similar to those observed in Parkinson’s disease remains largely unexplored.

Given the association between cadmium exposure and the development of PD, the aim of this study was to review intrinsic factors that could be associated with increased cadmium bioavailability and, consequently, an increased risk of PD.

## 2. Methods

We conducted a non-systematic, narrative literature review using the National Center for Biotechnology Information, Google Scholar, and PubMed databases. The following search terms were used: “Cadmium, Parkinson disease,” “Zinc deficiency and risk of Parkinson’s disease,” “COPD and risk of Parkinson’s disease,” “iron deficiency and risk of PD,” “cadmium neuronal toxicity,” “Cd exposure and gut microbiota,” and “Parkinson’s disease and gut microbiota.” No restrictions were applied regarding the publication date of the included studies. Mini-reviews and case reports were excluded. This manuscript represents a review and not an original research article.

### 2.1. Iron Deficiency Anemia, COPD, and Zinc Deficiency as Promoters of PD

Individuals living in the same area are typically exposed to cadmium through similar sources, as they consume similar food, breathe the same air, and drink the same water. However, despite these shared exposures, blood concentrations of cadmium and its bioavailability can vary significantly from person to person. This variation suggests that intrinsic factors exert influence on the concentration of cadmium entering the systemic circulation. An example of this is seen in individuals who have anemia (or iron deficiency—IDA), chronic obstructive pulmonary disease (COPD) and zinc deficiency. Iron deficiency and COPD have been linked to higher blood cadmium levels due to the overexpression of the intestinal iron transporter known as divalent metal transporter-1 (DMT-1), which is responsible not only for mediating iron transport but also for transporting other divalent ions, such as cadmium [29]. While it is well known that zinc plays an important role in inhibiting Cd absorption by competition, suggesting that zinc deficiency may be associated with higher Cd levels [30].

Nowadays, there is enough molecular evidence that Cd may utilize the in order to be transported via the intestinal barrier. Ohta and Ohba demonstrated that at least three different intestinal transporters—the iron transporter DMT-1, the zinc transporter ZIP14, and the copper transporter ATP7A—are capable of transporting Cd [31]. Because Cd shares similar physicochemical properties, including divalent ionic charge and comparable ionic radius (Cd and Ca in particular), with essential metals such as Fe, Zn, Cu, and Ca, it can compete for their intestinal transporters. These findings further suggest that either insufficient dietary intake of Fe, Cu, and Zn or systemic deficiencies of these metals may increase intestinal Cd uptake. This may occur because of reduced competition for the transporters in the setting of low oral intake, or because low circulating levels of Fe, Cu, and Zn induce overexpression of these transporters. In the case of DMT-1, evidence indicates that the transporter has a greater affinity for Cd than for Fe [32]. Also, Min et al. demonstrated that Cd can potentially utilize the calcium intestinal carrier [33]. Several intestinal metal transporters are capable of mediating Cd uptake. Although the roles of copper and calcium transporters in PD are beyond the scope of this paper, two independent studies reported that higher dietary intake of calcium [34] and copper [35] is associated with a lower risk of PD. This association may be explained either by increased Cd absorption in cases of calcium or copper deficiency/low intake, due to reduced competition for intestinal transporters, or by the absence of the potential neuroprotective effects of copper.

IDA, which affects over 1 billion individuals worldwide [36], also increases the risk of Parkinson’s disease (PD) [37]. In a study by Hong et al., slightly over 86,000 anemic individuals over the age of 45 years were compared to a similar number of non-anemic individuals. The study suggested that anemic individuals had a significantly higher risk of developing PD, and iron supplementation did not reduce this risk [37]. Additionally, Savica and colleagues found an association between the appearance of PD and anemia that occurred 2–3 decades prior [38]. Another study from Korea, performed by Kim et al., included slightly fewer than 30,000 individuals, of whom 5844 had PD and 23,376 did not. The study reported an increased risk of PD in the case of anemia, particularly among men under the age of 70 years [39]. Finally, Wang et al. conducted a meta-analysis that unambiguously confirmed the association between anemia and PD [40].

Chronic obstructive pulmonary disease represents another intrinsic factor associated with increasing Cd levels [29,41] and PD risk development. A large cohort study from Taiwan, which included nearly 21,000 individuals with COPD and 41,147 healthy controls, reported an almost 40% higher risk of PD in COPD individuals [42]. Literature indicates that COPD patients exposed to elevated levels of PM2.5 (airborne particulate matter that often contains cadmium [43]), have a greater risk of developing PD compared to non-exposed individuals [44]. Additionally, exposure to cadmium in PM2.5 can exacerbate specific behavioral symptoms in PD patients [45] since individuals living in PM2.5-polluted areas tend to have higher blood Cd concentrations [46]. Finally, zinc deficiency, a third intrinsic factor reviewed in our study, has been associated with cadmium toxicity, since low levels of zinc may increase Cd absorption and accumulation [30]. In a meta-analysis by Du et al., which included 23 original studies, it was revealed that Zinc blood deficiency has also been observed in patients with PD when compared to healthy control patients [47]. In this way, it is reasonable to speculate that Iron deficiency anemia, COPD, and zinc deficiency contribute to increased Cd blood levels, as we have detailed in our previous work [29,41] and could contribute to the onset and progression of PD.

### 2.2. Cd-Induced Brain Alteration Which Could Promote Onset of Parkinson’s Disease

It is reasonable to speculate that COPD and IDA not only promote cadmium intestinal absorption, but they also facilitate the uptake of heavy metals in peripheral tissues [48,49], including the brain [50]. Both COPD and IDA lead to generalized hypoxia and generalized hypoxic conditions in all tissues, particularly in severe forms of these diseases. As a compensatory mechanism, transferrin receptor 1 (TfR1) becomes overexpressed in all peripheral tissues because TfR1 is a hypoxia-inducible gene [51,52]. Cabrera et al. demonstrated that ID promoted the expression of TfR1 [53]. Cadmium is mainly bound to transferrin in the bloodstream, and the transferrin-TfR1 axis serves as the main pathway for both iron [54] and cadmium to enter the brain via the blood–brain barrier (BBB) [55]. To some extent, Cd also binds to hemoglobin [56] and albumin. Egger et al. measured Cd concentrations in 40 different human tissues from four individuals and confirmed the previously established finding that Cd accumulates at high concentrations in the liver and kidneys. Regarding neuronal tissue, the authors analyzed the cerebral cortex and reported Cd concentrations of 38.9 mg kg^−1^, 43.2 mg kg^−1^, 65.6 mg kg^−1^, and 13.2 mg kg^−1^. These findings unequivocally confirm that Cd crosses the blood–brain barrier (BBB) and accumulates in neuronal tissue at varying concentrations. Notably, analysis of only four cases revealed an almost fivefold difference in Cd concentration within the cerebral cortex, indicating substantial interindividual variability in cerebral Cd accumulation [57].

**Figure 1:**
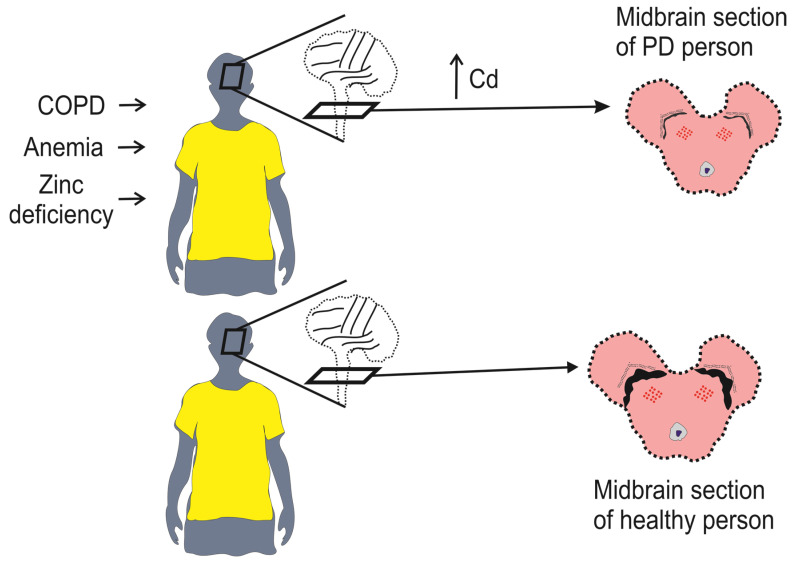
Schematic representation of the contribution of common comorbidities and conditions to the development of Parkinson’s disease. The upper section illustrates degeneration of the substantia nigra associated with increased cadmium accumulation, whereas the lower right section depicts a healthy midbrain with an intact substantia nigra. Loss of dopaminergic neurons in the substantia nigra leads to altered innervation of the corpus striatum, which represents a key pathophysiological feature of PD.

This mechanism could explain the delayed relationship between exhibiting anemia and the PD onset, as these two events could be separated by decades [38], in view of the fact that Cd accumulates gradually. Moreover, cadmium elimination from the body is an extremely slow process, with Cd remaining in the body for decades [58]. Cadmium exhibits its neurotoxicity through several mechanisms, primarily by promoting oxidative stress, altering mitochondrial function and interfering with normal glucose and neurotransmitter signaling processes [55]. Gupta et al. have shown that Cd could disrupt the dopaminergic system in an in vivo Wistar male rat model, with the test animals orally treated with cadmium for 28 days. The results showed a reduction in mRNA expression of DA-D2 receptors in the corpus striatum of cadmium-exposed rats, followed by a loss of striatal neurons [59]. Similarly, Xu et al. conducted a study using 8-week-old male C57BL/6 mice orally exposed to Cd. Behavioral assessments revealed that the Cd-exposed mice were less active, displaying prolonged periods of immobility [60]. Xu et al. also found increased Cd levels in the midbrains of Cd-treated mice [60] and identified several pathohistological alterations associated with Cd-induced neurotoxicity, such as axonal damage, a loss of TH-positive dopaminergic neurons in the substantia nigra compacta and a reduced number of neuronal spines (Figure 1). Further Cd treatment caused shifts and changes in the lipid patterns of substantia nigra neurons, notably showing a high frequency of proinflammatory lipids such as ceramides. In the second part of their study, involving human subjects, Xu et al. analyzed serum from PD patients and found similar changes regarding serum sphingolipid disturbances. Also, part of the study in which humans were involved revealed that serum Cd concentration was positively associated with the Unified Parkinson’s Disease rating scale score. Yang et al. measured Cd blood levels in almost 700 undergraduate students and demonstrated that Cd blood levels inversely correlated with tyrosine and levodopa, which are precursors for the synthesis of dopamine [61]. Two independent, in vivo studies revealed that exposure to Cd was associated with reduced concentrations of dopamine in the striatum [62] and hypothalamus [63] of treated rats. Taken together, those studies may support the hypothesis that Cd-induced neurotoxicity could be involved in the genesis of PD. The next “puzzle” in the pathophysiology of PD suggests that a lack of DA reduces the density of striatal spines (both types, mushroom and thin), which is a well-known feature of PD subjects [63]. Furthermore, the deficiency of DA interferes with excitability, leading to an increased firing rate in medium spiny neurons of both D1 and D2 types. Finally, the lack of DA results in a reduced amplitude of evoked excitatory postsynaptic potentials (EPSPs), selectively in D2-medium spiny neurons [64].

Luo et al. examined the potential association between Cd and NfL, a well-known marker of neurodegeneration [65,66]. They have analyzed 1040 participants and reported a linear dose-effect relationship between blood Cd and NfL serum levels [65]. On the other hand, Chen et al. conducted a cross-sectional study that included 170 individuals with PD and healthy controls, demonstrating that individuals with PD exhibited elevated blood concentrations of α-synuclein and NfL [67]. Another study with a larger sample size, consisting of 144 healthy controls and 301 de novo PD patients, showed higher serum NfL levels in PD patients when compared to controls [68].

### 2.3. FGF-23 as a Promoter of Brain Alterations and Vitamin D Deficiency in Patients with Parkinson’s Disease

FGF-23 is a protein with hormonal activity, primarily secreted by bone cells such as osteocytes and osteoblasts [69]. FGF-23 lowers blood phosphate levels and interferes with vitamin D synthesis [70]. Low hemoglobin concentrations are associated with increased circulating levels of the intact form of FGF-23 [71,72], which may explain why individuals with anemia often have vitamin D deficiency (VDD) [73]. Moreover, FGF-23 is elevated in individuals with COPD [74]. Also, Cd exposure rises FGF-23 concentrations [75]. Therefore, both comorbidities that increase the risk of PD also promote an increase in FGF-23 levels. Vitamin D deficiency is also a risk factor for the onset of Parkinson’s disease, with one in five PD patients having VDD [76]. Interestingly, individuals with PD have lower phosphate levels compared to controls [77]. On the one hand, FGF-23 reduces vitamin D levels, leading to vitamin D deficiency, which is a risk factor for PD [76]. On the other hand, elevated FGF-23 levels are associated with several brain alterations, including white matter injury, cognitive decline, and stenosis and calcification of brain arteries [78,79,80]. Additionally, the appearance of white matter impairments and the appearance of brain calcification are suggested to be more common in PD individuals [81]; furthermore [82], cognitive decline is a major non-motor symptom of PD [83].

In summary, Cd exposure, together with IDA and COPD, may promote FGF-23 synthesis, which in turn reduces vitamin D levels. VDD, independently, may further contribute to PD development. In addition, elevated FGF-23 levels have been associated with pathological features frequently observed in PD patients, including cognitive decline and white matter abnormalities.

### 2.4. Similarities Between Cd-Induced Changes in Gut Microbiota and Gut Microbiota in PD Patients

**Figure 2:**
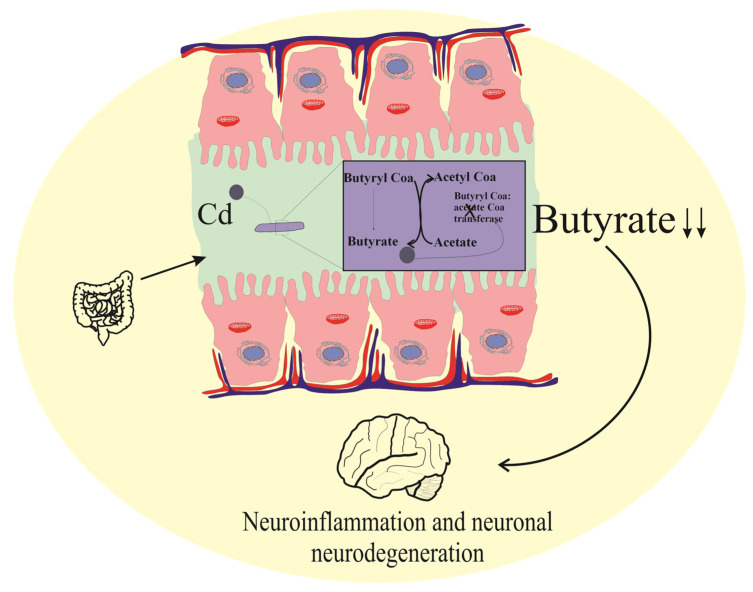
Cadmium in the intestine blocks the activity of Butyryl Coa: acetate Coa transferase and decreases butyrate production in the intestinal flora.

Keshavarzian et al. reported a decrease in the prevalence of some bacteria, such as Lachnospiraceae and Roseburia, in fecal samples of 38 PD patients when compared to 34 healthy controls [84]. Hill-Burns et al. analyzed gut microbiota in fecal samples obtained from 197 individuals diagnosed with PD and 130 healthy controls and reported a reduction in Lachnospiraceae [85]. Pietrucci et al. evaluated fecal microbiota composition in 80 patients with Parkinson’s disease and 72 healthy controls and reported lower levels of Lachnospiraceae in patients with Parkinson’s disease, as had been previously shown in the literature. Moreover, Pietrucci et al. noted that there is a significant correlation between lower levels of Lachnospiraceae and severity of motor impairment [86]. Barichella et al. collected over 190 fecal samples from patients with Parkinson’s disease and over 110 controls and specifically documented that lower abundance in Lachnospiraceae relative to controls was a specific feature of PD. Additionally, lower abundance in Lachnospiraceae correlated with cognitive decrease and motor impairment [87]. Breton et al. performed an in vivo study, in which they orally treated Balb/C female mice with cadmium and demonstrated lower abundance in Lachnospiraceae [88]. Butyrate-producing Lachnospiraceae generate butyrate, which in turn promotes the integrity of the intestinal barrier [89]. Cd can reduce the abundance of butyrate-producing Lachnospiraceae, and consequently, the intestinal barrier could gradually weaken its integrity. A compromised intestinal epithelial barrier is observed in patients with Parkinson’s disease [90]. Lachnospiraceae is among the bacterial families that are significant sources of butyrate producers [91,92]. Elford et al. stated that butyrate levels can have a critical role in the onset and progression of PD [93]. Cadmium has inhibitory effects on enzymes involved in butyrate synthesis [94] and, therefore, can powerfully deplete butyrate levels. Lower butyrate levels are observed in patients with Parkinson’s disease compared to controls [95] (Figure 2). Butyrate can modulate gene expression in the brain and has protective effects against neurodegeneration [96,97].

## 3. What About Other Metals?

As a concept, we provided an explanation of how increased cadmium load induced by COPD, IDA and zinc deficiency may be partly responsible for the genesis of PD. Nevertheless, isolated exposure to Cd may be seen only in in vivo studies, while humans are on a daily routine “in contact” with a greater number of metals. Therefore, other metals may contribute to the onset of PD as well. An example is Pb exposure, which has also been linked with PD onset [98]. Similarly, to Cd, Pb is also found in elevated levels in individuals with IDA [99]. Pb (II) and Fe (II) are absorbed from ingested food in their divalent forms. Increased Pb concentration in bones is associated with increased risk of PD onset [100]. An interesting study from India, which included 72 children aged 6–15 years, reported an inverse association between lead plasma concentrations and serum serotonin levels. Authors also demonstrated the effects of low and high plasma Cd concentrations. Participants (*n* = 72) were divided by high and low plasma lead levels. Based on median blood lead level (4.9 μg/dL), Malavika L. et al. showed that serum serotonin levels were significantly lower, while expression of serotonin receptor (5 H TR3A) was significantly higher in the group with higher lead blood levels. Other studies revealed that patients with PD exhibit reduced serotonin levels in cerebrospinal fluid and the caudate nucleus [101,102]. Next, dopamine receptor (DRD2 and DRD3) expression was altered in the group of children with increased blood lead levels [103]. Malavika L. also noted that children with higher blood levels of lead were prone to anxiety, depression, behavioral problems and physical illness with emotional problems as well [103]. Serum iron levels were found to be elevated in PD subjects compared to healthy controls [100]. It is reasonable to speculate that iron, Cd and Pb, which are transported by Tf, accumulate in peripheral tissues, including the brain, in the case of COPD and IDA. As a limitation, we must note that PD is a complex, multifactorial disease with a genetic background, and metal exposure may be considered as a contributing factor.

## 4. Limitations and Final Discussion

In this manuscript, we did not discuss all contributing mechanisms that may enhance the onset of Parkinson’s disease. The main goal of this paper is to draw attention to the possibility that environmental factors, such as Cd exposure, may promote PD development in a particular subset of individuals with COPD, ID, and zinc deficiency, representing an initial step toward disease onset. Consequently, over time, these individuals may gradually accumulate Cd through the intestinal tract if COPD, ID, and zinc deficiency are not adequately treated. If they smoke, the situation may become even worse, as cigarettes are also a significant source of Cd exposure. Increased Cd burden, as well as COPD independently, may elevate FGF-23 levels and thereby decrease vitamin D concentrations. Cadmium exerts direct neurotoxic effects, while vitamin D deficiency may further contribute to PD development. If this pathological mechanism could be divided into stages, the initial phase would likely involve enhanced Cd accumulation due to COPD, ID, zinc deficiency, and smoking, followed by progressive neuronal damage mediated by Cd toxicity, vitamin D deficiency, and chronic systemic disturbances. In parallel with the gradual increase in Cd burden, intestinal Cd exposure may adversely affect the gut microbiota and induce changes similar to those observed in PD. These Cd-induced alterations of the intestinal microbiota may further contribute to disease onset.

Importantly, the proposed relationship between iron deficiency anemia, COPD, zinc deficiency, increased cadmium burden, and Parkinson’s disease should be considered hypothetical and potentially influenced by multiple confounding factors. Smoking is particularly important because it represents a major independent source of cadmium exposure and is strongly associated with COPD. Likewise, air pollution and PM2.5 exposure may simultaneously contribute to pulmonary disease, systemic inflammation, cadmium exposure, and neurodegeneration. Occupational exposure, dietary habits, renal function, and age may also substantially influence cadmium absorption, distribution, accumulation, and elimination. Therefore, the mechanisms proposed in this review should be interpreted cautiously until validated by prospective clinical and mechanistic studies specifically designed to control for these confounders.

## 5. Conclusions

Cadmium is a neurotoxic metal that, over chronic exposure, may contribute to the progression of Parkinson’s disease. Cadmium adverse effects are dose and time-dependent, and cadmium in subacute exposure causes a gradual loss of neurons within the substantia nigra. This gradual loss of neurons fits with the fact that a significant part of the PD neurodegenerative process is asymptomatic and takes years prior to the onset of symptoms. In that sense, gradual Cd-induced neurotoxicity could provide a reasonable explanation for the onset and progression of the pathology. Although Cd is not the sole agent responsible for PD onset—since PD is a multifactorial disease—it can be considered a powerful contributing factor. The main message of this paper is that neurologists should be aware that patients with PD and comorbid conditions like iron deficiency anemia, COPD, or zinc deficiency are likely to have elevated blood concentrations of Cd, which has potent pro-parkinsonian effects. Cd exposure decreases the expression of D2 receptors in the neostriatum and leads to the generation of pro-inflammatory lipids in the midbrain. NfL has been found to be elevated in PD patients and correlates with blood cadmium levels. Increased levels of FGF-23, frequently found in ID and COPD subjects, promote the appearance of VDD, which raises the risk of PD onset. Cadmium is capable of inducing intestinal dysbiosis and a reduction in butyrate production in an analogous manner as observed in PD subjects, indicating that Cd can promote PD genesis by causing disturbances in non-neural tissue.

## Data Availability

No new data were created or analyzed in this study.

## Abbreviations

Cd	Cadmium
COPD	Chronic Obstructive Pulmonary Disease
DA	Dopamine
DMT1	Divalent Metal Transporter 1
EPSPs	Excitatory Postsynaptic Potentials
FGF-23	Fibroblast Growth Factor 23
ID	Iron Deficiency
IDA	Iron Deficiency Anemia
NfL	Neurofilament Light Chain
Pb	Lead
PD	Parkinson’s Disease
PM2.5	Particulate Matter ≤ 2.5 μm in diameter
TH	Tyrosine Hydroxylase
Tf	Transferrin
TfR1	Transferrin Receptor 1
UPDRS	Unified Parkinson’s Disease Rating Scale
VDD	Vitamin D Deficiency
